# The relationship of α-hydroxybutyrate dehydrogenase with 1-year outcomes in patients with intracerebral hemorrhage: A retrospective study

**DOI:** 10.3389/fneur.2022.906249

**Published:** 2022-10-18

**Authors:** Zhang Limin, Rasha Alsamani, Wu Jianwei, Shi Yijun, Wang Dan, Sun Yuehong, Liu Ziwei, Xu Huiwen, Wang Dongzhi, Zhao Xingquan, Zhang Guojun

**Affiliations:** ^1^Department of Clinical Diagnosis Laboratory of Beijing Tiantan Hospital, Capital Medical University, Beijing, China; ^2^NMPA Key Laboratory for Quality Control of In Vitro Diagnostics, Beijing, China; ^3^Beijing Engineering Research Center of Immunological Reagents Clinical Research, Beijing, China; ^4^Department of Neurology, Beijing Tiantan Hospital, Capital Medical University, Beijing, China

**Keywords:** intracerebral hemorrhage, outcomes, mortality, α-HBDH, cardiovascular events

## Abstract

**Background and aims:**

Cardiac enzymes are recognized as a valuable tool for predicting the prognosis of various cardiovascular diseases. The prognostic value of alpha-hydroxybutyrate dehydrogenase (α-HBDH) in patients with intracerebral hemorrhage (ICH) was ambiguous and not evaluated.

**Methods:**

Two hundred and thirteen Chinese patients with ICH participated in the study from December 2018 to December 2019. Laboratory routine tests and cardiac enzymes, including α-HBDH level, were examined and analyzed. All the patients were classified into two groups by the median value of α-HBDH: B1 <175.90 and B2 ≥175.90 U/L. The clinical outcomes included functional outcome (according to modified Rankin Scale (mRS) score ≥3), all-cause death, and recurrent cerebro-cardiovascular events 1 year after discharge. Associations between the α-HBDH and the outcomes were evaluated using logistic regression analysis. Univariate survival analysis was performed by the Kaplan–Meier method and log-rank test.

**Results:**

Of the 213 patients, 117 had α-HBDH ≥175.90 U/L. Eighty-two patients had poor functional outcomes (mRS≥3). During the 1-year follow-up, a total of 20 patients died, and 15 of them had α-HBDH ≥175.90 U/L during the follow-up time. Moreover, 24 recurrent events were recorded. After adjusting confounding factors, α-HBDH (≥175.90) remained an indicator of poor outcome (mRS 3-6), all-cause death, and recurrent cerebro-cardiovascular events. The ORs for B2 vs. B1 were 4.78 (95% CI: 2.60 to 8.78, *P* = 0.001), 2.63 (95% CI: 0.80 to 8.59, *P* = 0.11), and 2.40 (95% CI: 0.82 to 7.02, *P* = 0.11) for poor functional outcomes with mRS ≥ 3, all-cause death, and recurrent cerebro-cardiovascular events, respectively.

**Conclusion:**

Increased α-HBDH at admission was independently related to poor functional outcome and all-cause mortality in patients with ICH at 1-year follow-up.

## Introduction

In recent years, the stroke burden in China has increased. A survey reported that the prevalence of stroke in China has continued to increase in the past 7 years (2013–2019) (1). Intracerebral hemorrhage (ICH) is a life-threatening subtype of stroke, which affects about 2 million people worldwide each year (2, 3). Moreover, the therapies of ICH remain restricted, despite unceasing efforts to find efficient management (4).

Cardiovascular complications are associated with an increased risk of death and a poor prognosis in ICH (5). Within 2 days after a stroke, patients with ICH can develop severe cardiac disorders such as ventricular arrhythmias, cardiomyopathy, and heart failure (6). In contrast, there is no evidence that ICH causes severe and chronic or sudden myocardial damage without relevant heart disease. The processes that underlie ICH-induced cardiac dysfunction are still unknown. Cardiac enzymes are recognized as valuable tools for predicting the presence or prognosis of various cardiovascular diseases (7). Based on previous studies, high myoglobin (MYO) level has been found in ICH and ischemic stroke (8, 9). A retrospective study reported that serum cardiac troponin I (cTnI) level can predict adverse outcomes and mortality in ICH (10). Therefore, cardiac biomarkers are vital for indicating cardiovascular abnormalities in the post-stroke period for better diagnosis and better management of these complications. Alpha-hydroxybutyrate dehydrogenase (α-HBDH) is a cardiac enzyme commonly found in the heart, brain, kidneys, and red blood cells (11). Elevated α-HBDH has been reported to be related to myocardial infarction and hepatic damage (12). Recent research has revealed that serum α-HBDH levels have a role in atherothrombotic events (13). However, the significance of α-HBDH in stroke is still unclear.

It has not been reported if there are any significant changes in circulating α-HBDH levels in ICH or whether they have predictive value for ICH outcomes. Hence, we investigated the relation between α-HBDH and functional outcomes, all-cause mortality, and recurrence of cerebro-cardiovascular events in patients with ICH after 1 year of discharge.

## Materials and methods

### Patient enrollment

This study was a retrospective, observational study. The Ethics Committee of Beijing Tiantan Hospital of Capital Medical University, China (KY 2020-076-02), approved the study protocol by the Declaration of Helsinki.

We enrolled consecutive patients with ICH older than 18 years who presented directly to Beijing Tiantan Hospital of Capital Medical University at the emergency department between December 2018 and December 2019. The patients included had a baseline NCCT scan within 24 h of ICH onset and a follow-up NCCT scan within 48 h of the initial CT scan. Patients diagnosed with arteriovenous malformation, cerebral aneurysm, moyamoya syndrome, brain tumor, or hematonosis and undertaken surgical operation before follow-up NCCT scan were excluded from this study. Furthermore, hemorrhagic cerebral infarction, bleeding caused by trauma, or hemostatic treatment of ischemic stroke while hospitalized were excluded either.

**Figure 1:**
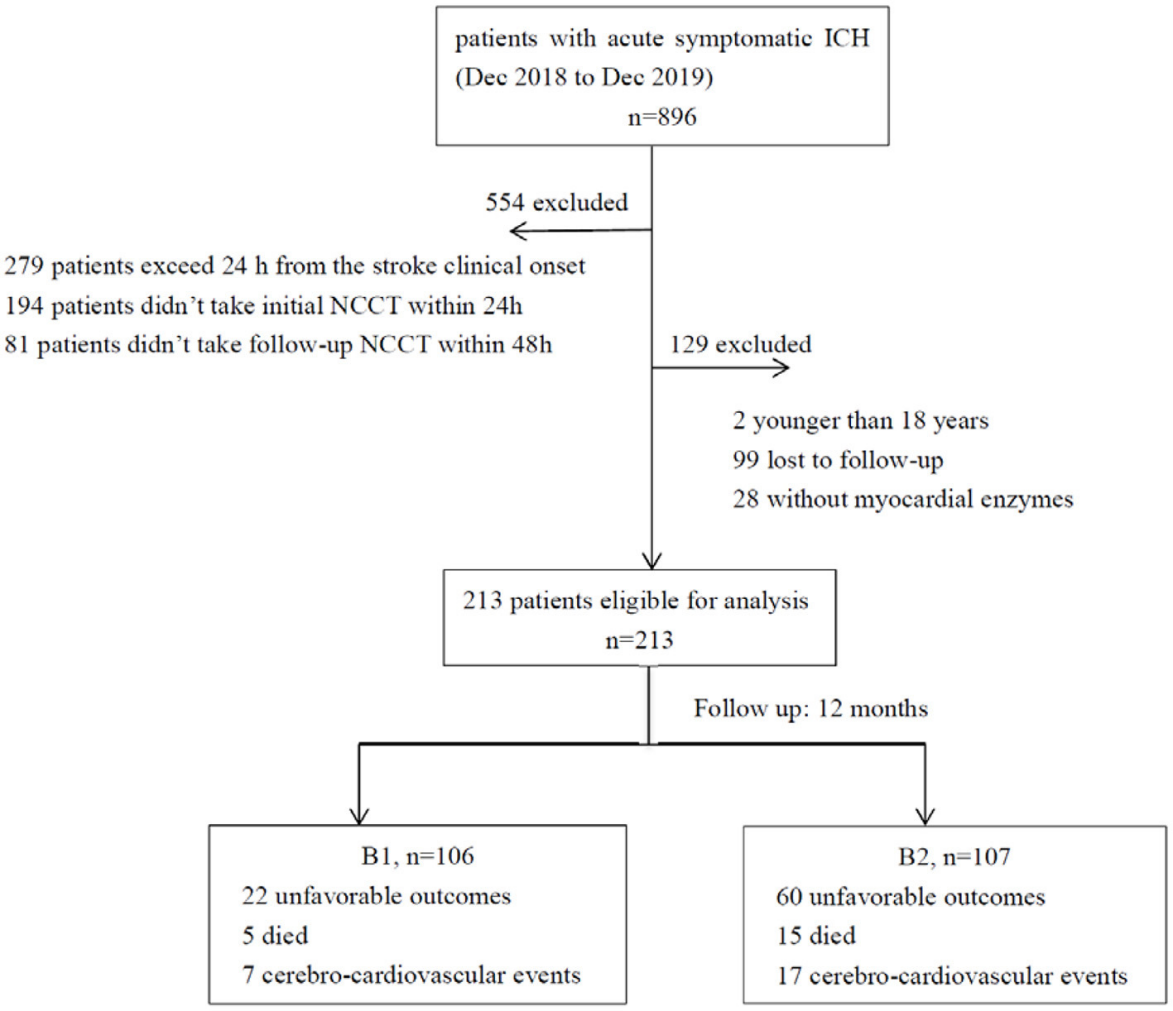
Enrollment flow chart for analysis. ICH, Intracerebral hemorrhage; NCCT, non-contrast CT.

At first, 896 patients with ICH were enrolled. We excluded patients who were more than 24 h after the clinical stroke onset (*n* = 279), did not take the initial NCCT within 24 h (*n* = 194), or did not take the follow-up NCCT within 48 h (*n* = 81). We also excluded 2 patients under 18 years, 99 who did not have follow-up records, and 28 who did not have α-HBDH values. Finally, in this study, 213 patients with ICH were examined (Figure 1).

### Baseline information

For each patient, trained neurological physicians collected comprehensive information, including gender, age, history of hypertension, hyperglycemia, dyslipidemia, cigarettes, alcohol intake, and prescribed medications (such as antihypertensive/antiplatelet medications). National Institutes of Health Stroke Scale (NIHSS) scores, initial CT scan, and time of ICH onset were recorded after admission. On the initial CT scan, the bleeding site, presence of intraventricular hemorrhage (IVH), and hematoma volume were evaluated by us. The bleeding sites were divided into basal ganglia, cerebellum, lobar, and brainstem. The volume of hematoma was determined by the ABC/2 method (14).

### Evaluation *t* of α-HBDH level and other laboratory examinations

Fasting blood samples were gathered and analyzed within 24 h of admission. The α-HBDH concentration was determined using an enzyme kinetic method and an α-hydroxybutyrate dehydrogenase kit. The instrument used in the experiment was an automated biochemical immunoassay analyzer (LABOSPECT 008 AS).

The reference concentration of α-HBDH level was 72–182 U/L. In addition, tests for brain natriuretic peptide (BNP), alanine transaminase (ALT), MYO, cTnI, creatine kinase (CK), aspartate transaminase (AST), and white blood cell counts (WBC) were performed.

### Follow-up information

The follow-up information was obtained through clinic or telephone after 1-year follow-up. The modified Rankin scale (mRS) was used to evaluate the therapeutic effect. The mRS score≥3 indicated a poor functional prognosis (15). During the follow-up evaluation, the interviewers were unaware of any prognostic factors and were trained on the interview protocol. Adverse outcomes include poor functional outcomes, all-cause mortality, and recurrence of cerebro-cardiovascular events. Death from any cause was confirmed by a death certificate issued by a hospital or registrar of residents in the area where the patient was treated. Cerebro-cardiovascular events were defined as acute myocardial infarction, acute heart failure, and recurrent stroke, which were checked in the medical records from the attending hospital.

### Statistical analysis

Patients were classified into two groups according to the median of α-HBDH: B1 <175.90 and B2 ≥175.90 U/L. The results were expressed as number and percentage for categorical variables, mean and standard deviation (SD) for normally distributed continuous variables, and medians and interquartile ranges for continuous variables not normally distributed. Chi-squared, Student's *t*-tests, or Wilcoxon rank-sum tests were used to assess differences in categorical or continuous factors among different patient categories based on the α-HBDH.

The relation of α-HBDH with functional outcomes, all-cause mortality, and recurrence of cerebro-cardiovascular events were assessed by logistic regressions. The variables with *P* < 0.1 in the univariate analysis and the risk factors for poor outcomes were chosen. We also estimated unadjusted and adjusted odds ratios (ORs) and their 95% confidence intervals (CIs). Model 1 was adjusted for age and gender; Model 2 was additionally adjusted for BMI, smoker, alcohol, SBP, history of high blood pressure, dyslipidemia, diabetes and coronary artery disease, hematoma volume, and bleeding site; Model 3 was further adjusted for ALT, AST, CK, CK-MB, MYO, cTnI, and WBC on admission. The univariate survival analysis was compared by Kaplan–Meier analysis and the log-rank testing. Log-rank test was used to evaluate the difference in survival probability between groups.

Statistical analyses were performed using the SPSS statistical package version 23.0 (SPSS Inc., Chicago, USA) and R software, version 3.3.1 (http://www.R-project.org/). All statistical tests were two-tailed, and a *P*-value < 0.05 was considered statistically significant.

## Results

### Baseline characteristics of included patients according to the median of α-HBDH

A total of 213 primary ICH patients with an average age of 54.31 ± 13.38 years were recruited for this study (Figure 1). The median (IQR) α-HBDH of all the patients was 175.90 (155.30, 210.95) U/L, and the mean ± SD value was 188.47 ± 53.97U/L. One hundred and seven patients had α-HBDH ≥175.90 U/L. The general characteristics of patients and groups based on the α-HBDH level are shown in Table 1.

**Table 1:** Baseline characteristics of all patients grouped by α- HBDH levels.

**Variables**	α**- HBDH(U/L)**			***P-*value**
	**All** **(*n =* 213)**	** < 175.90** **(*n =* 106)**	**≥175.90** **(*n =* 107)**	
Male gender, *n* (%)	174(81.7)	91(85.8)	83(77.6)	0.118
Age, year	54.31 ± 13.38	54.12 ± 11.44	54.50 ± 15.11	0.835
BMI	25.83(23.66,27.76)	25.95 (23.50,27.75)	25.30(23.71,27.76)	0.879
Smoking history	99	53(50.0)	46(43.0)	0.305
Alcohol history	122(57.3)	65(61.3)	57(53.3)	0.235
History of hypertension	156(73.2)	76(71.7)	80(74.8)	0.613
History of dyslipidemia	43(20.2)	21(19.8)	22(20.6)	0.892
History of diabetes	35(16.4)	20(18.9)	15(14.0)	0.340
Coronary heart disease	23(10.8)	12(11.3)	11(10.3)	0.807
Systolic pressure(mmHg)	161.62 ± 23.31	159.46 ± 23.14	163.76 ± 23.39	0.179
Diastolic pressure(mmHg)	97.69 ± 17.21	97.90 ± 16.15	97.50 ± 18.27	0.866
Antihypertensive medication	71(33.3)	32(30.2)	39(36.4)	0.333
Lipid-lowering medicine	21(9.9)	13(12.3)	8(7.5)	0.241
antidiabetic	17(8.0)	8(7.5)	9(8.4)	0.816
**Antiplatelet medication**	17(8.0)	8(7.5)	9(8.4)	0.816
Anticoagulants *n* (%)	8(3.8)	4(3.8)	4(3.7)	1.000
Time from onset to initial NCCT (h)	6(3,8)	5.0(2.5,7.25)	6(3,9)	0.265
NIHSS score	7 (4,12)	6(3,10)	8(5,13)	**0.003**
Baseline hematoma volume (ml)	20.00(9.31,34.10)	16.09(7.93,25.65)	25.80 (10.80,45.00)	**0.001**
Hematoma expansion, *n* (%)	9(6.5)	2(1.9)	7(6.5)	0.170
**Hematoma location**, ***n*** **(%)**				0.089
Deep	137(64.3)	69(65.1)	68(63.6)	
Lobar	44(20.6)	19(17.9)	25(23.3)	
Infratentorial	26(12.2)	16(15.1)	10(9.4)	
IVH	6(2.8)	2(1.9)	4(3.7)	
mRS 3-6 at 1 year, *n* (%)	82(38.50)	22(20.8)	60(56.1)	**0.000**
Recurrence of cerebro-cardiovascular events at 1 year, *n* (%)	24(11.3)	7(6.6)	17(15.9)	**0.032**
All-cause Mortality, *n* (%)	20(9.4)	5(4.7)	15(14.0)	**0.020**
BNP(pg/ml)	32.20(15.40,69.35)	30.90(14.70,66.00)	34.10(16.65,78.95)	0.639
cTnI(ng/ml)	0.004(0.002,0.010)	0.003(0.002,0.007)	0.005(0.002,0.155)	**0.049**
MYO(ng/ml)	51.65(31.78,93.10)	43.90(30.55,75.20)	60.50(35.70,125.70)	**0.011**
ALT(U/L)	19.30(14.00,28.40)	17.70(13.45,24.43)	20.10(15.20,33.00)	**0.033**
AST(U/L)	19.50(16.00,25.55)	17.65(14.28,22.00)	21.80(18.20,30.00)	**0.000**
CK(U/L)	76.80(51.75,147.25)	67.25(40.80,105.95)	96.30(60.30,184.00)	**0.000**
CK-MB(U/L)	13.35(10.93,16.98)	12.35(10.30,15.98)	14.45(12.18,18.60)	**0.000**
WBC(*10^9^/L)	9.18(7.35,11.42)	8.69(6.78,10.91)	9.71(7.78,12.70)	**0.011**

Data are expressed as the mean ± standard deviation (SD), median (IQR), or n (%) as appropriate.

NIHSS, National Institutes of Health Stroke Scale; IVH, intraventricular hemorrhage; NCCT, non-contrast CT; α-HBDH, α-hydroxybutyrate dehydrogenase; BNP, brain natriuretic peptide; cTnI, cardiac troponin I; MYO, myoglobin; ALT, alanine aminotransferase; AST, aspartate aminotransferase; CK, creatine kinase; CK-MB, creatine kinase-MB; WBC, white blood cells.

The median NIHSS score was 7 for the entire cohort (4, 12). At baseline, the median hematoma volume was 20.00 (9.13, 34.10) ml. There were 137 patients (64.3%) whose hematomas were located in the supratentorial deep gray matter and 44 patients (20.6%) whose hematomas were accompanied by lobar locations. Nine patients (6.5%) had hematoma growth. Eighty-two patients (38.50% of the cohort) had poor functional outcomes (mRS Score ≥3) at 1-year follow-up.

**Figure 2:**
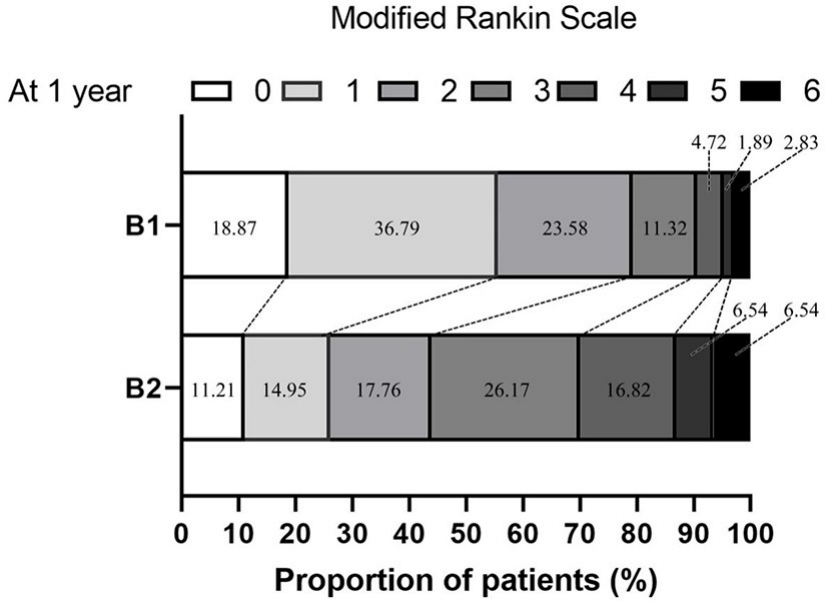
The mRS scores distribution stratified by median α-HBDH.

The mRS scores distribution stratified by median α-HBDH were shown in Figure 2. There was a high proportion of mRS scores of 3 to 6 in the B2 group compared with B1. The number of all-cause death during the follow-up was 20. Fifteen of the 20 patients were from the B2 group with α-HBDH≥ 175.90 U/L. Of the 20 deaths, 10 were caused by hemorrhagic stroke, 2 ischemic strokes, 2 acute myocardial infarctions, and 1 heart failure. Of the other five deaths, two were due to severe pulmonary infection and three were caused by other reasons, respectively.

Twenty-four patients experienced recurrent cerebro-cardiovascular events, including 19 new strokes, hemorrhagic or ischemic, three acute myocardial infarctions, and two heart failures. Seventeen of the 24 cases were from the B2 group.

Regarding markers of myocardial injury, significant intergroup differences were noted for cTnI, MYO, ALT (P <0.05), AST, CK, and CK-MB (*P* < 0.001). The B2 group had a high level of WBC compared with B1 (*P* = 0.011).

### The correlation of markers of myocardial injury and WBC

As shown in Table 2, the α-HBDH level was linked to ALT, AST, CK, CK-MB, and MYO (*r* = 0.216, 0.379, 0.254, 0.352, and 0.235, respectively). On the other hand, the WBC was correlated with α-HBDH, CK-MB, MYO, and cTnI (*r* = 0.213, 0.338, 0.282, and 0.357, respectively).

**Table 2:** Correlation between α-HBDH and myocardial enzymes and inflammation.

	**α-HBDH**	**ALT**	**AST**	**CK**	**CK-MB**	**MYO**	**cTnI**	**BNP**
ALT	0.216** 0.002							
AST	0.379** 0.000	0.540** 0.000						
CK	0.254** 0.000	0.004 0.951	0.207** 0.000					
CK-MB	0.352** 0.000	0.066 0.341	0.190** 0.005	0.466** 0.000				
MYO	0.235** 0.001	0.001 0.983	0.177* 0.012	0.358** 0.000	0.373** 0.000			
cTnI	0.093 0.189	−0.033 0.642	0.010 0.886	0.046 0.518	0.439** 0.000	0.371** 0.000		
BNP	0.053 0.447	−0.091 0.190	−0.011 0.875	0.103 0.141	0.236** 0.000	0.325* 0.000	0.439** 0.000	
WBC	0.213** 0.002	0.055 0.428	0.012 0.867	0.060 0.384	0.338** 0.000	0.282** 0.000	0.357** 0.000	0.113 0.105

α-HBDH, hydroxybutyrate dehydrogenase; ALT, alanine aminotransferase; AST, aspartate aminotransferase; CK, creatine kinase; CK-MB, creatine kinase-MB; MYO, myoglobin; cTnI, cardiac troponin I; BNP, brain natriuretic peptide; WBC, white blood cells.

^**^Correlation is significant at the 0.01 level (two-tailed).

^*^Correlation is significant at the 0.05 level (two-tailed).

### Association between α-HBDH and functional outcome, all-cause mortality, and recurrent cerebro-cardiovascular events

**Figure 3:**
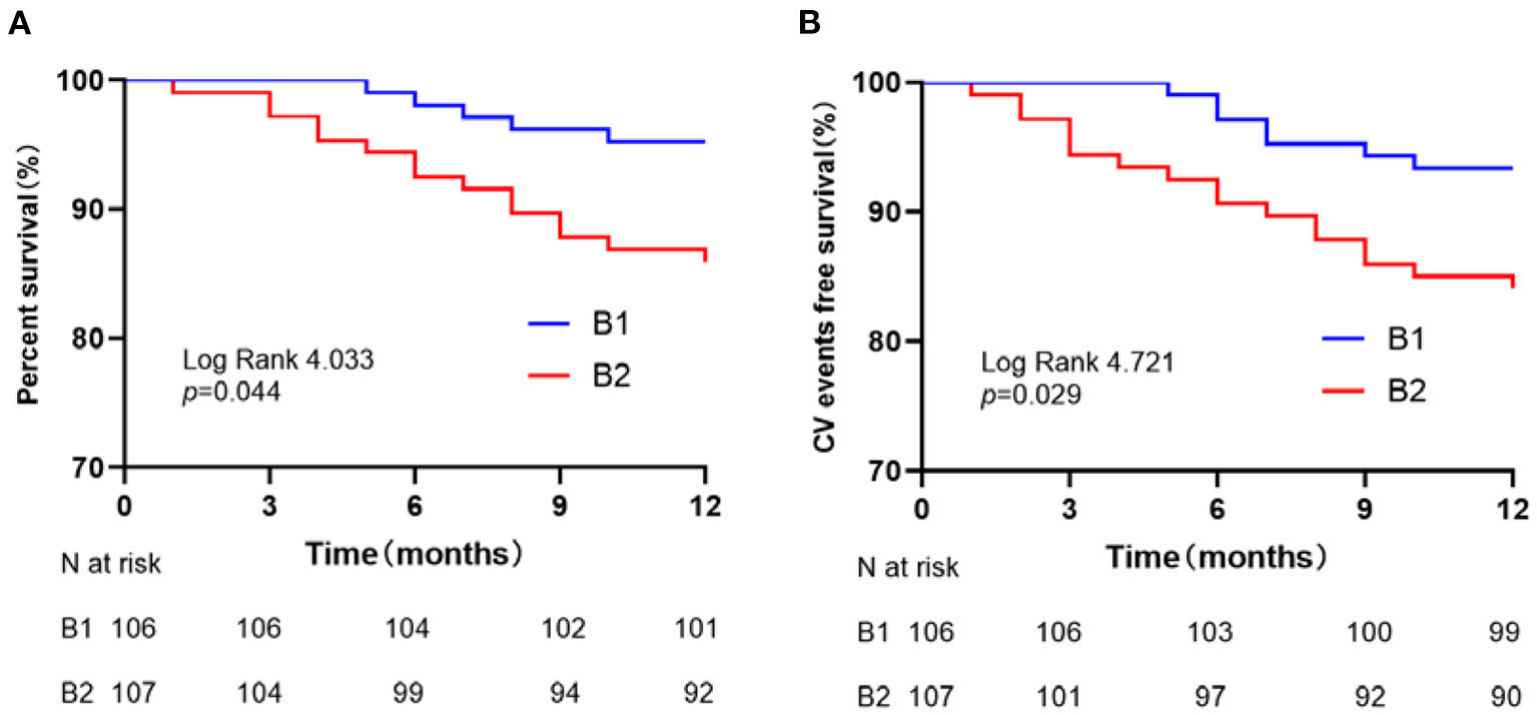
Survival curves for patients stratified by α-HBDH. **(A)** All-cause mortality-free survival curves. **(B)** Cerebro-cardiovascular events-free survival curves.

To see the influence of α-HBDH levels on clinical outcomes, logistic regression analysis was performed with a higher median HBDH level (Table 3). A significant association was found between high α-HBDH levels and clinical outcomes when 175.9 was used as the cut-off value. In Table 3, although Models 1 and 2 had been adjusted, the α-HBDH was still associated with poor functional outcomes (mRS 3–6), all-cause mortality, and recurrent cerebro-cardiovascular events. Moreover, the result also suggested that α-HBDH was correlated with functional outcome (*P* < 0.001, O*R* = 4.78, 95% CI: 2.60 to 8.78) in Model 3 even after adjusting for other laboratory confounding factors. While in Model 3, the ORs for B2 vs. B1 were 2.63 (95% CI: 0.80 to 8.59, *P*-value = 0.11) and 2.40 (95% CI: 0.82 to 7.02, *P*-value = 0.11) for all-cause mortality and recurrent cerebro-cardiovascular events, respectively. The Kaplan–Meier curves with median α**-**HBDH showed early separation and continued to diverge throughout the follow-up period (Figures 3A,B). The survival rates assessed at the end of 3, 6, and 12 months were 100, 98.1, and 95.3% in the B1 group, respectively. Meanwhile, the rates in the B2 group were 97.2, 92.5, and 86.0%, respectively (Figure 3A). At the end of 3, 6, and 12 months, the recurrence of the cerebro-cardiovascular events was 0, 2.8%, and 6.6% in the B1 group. The rates in the B2 group were 5.6, 9.3, and 15.9%, respectively (Figure 3B). Patients with a high level of α-HBDH showed a higher cumulative incidence of all-cause death and cerebro-cardiovascular events recurrence at 1 year (log-rank test *P* = 0.044 and 0.029, respectively).

**Table 3:** Associations of poor functional outcome, all-cause mortality, and cerebro-cardiovascular events with a median of α-HBDH (B2 vs. B1).

**Outcomes**	**Events** ***n* (%)**	**Unadjusted**	**Model 1** ** OR (95%CI)**	**Model 2** ** OR (95%CI)**	**Model 3** ** OR (95%CI)**
**mRS 3-6**	82(38.50)				
B1	22(10.33)	Reference group	Reference group	Reference group	Reference group
B2	60(28.17)	4.87(2.66,8.93)	4.78(2.60,8.78)	5.09(2.44,10.67)	4.77(2.10,10.84)
*P*-value		**< 0.001**	**< 0.001**	**< 0.001**	**< 0.001**
**All-cause mortality**	20(0.4)				
B1	5(4.7)	Reference group	Reference group	Reference group	Reference group
B2	15(7.04)	3.29(1.15,9.42)	3.12(1.08,8.98)	3.55(1.17,10.76)	2.63(0.80,8.59)
*P-*value		**0.026**	**0.035**	**0.025**	**0.11**
**Recurrentcerebro-cardiovascular events**	24(11.3)				
B1	7(6.6)	Reference group	Reference group	Reference group	Reference group
B2	17(15.9)	2.67(1.06,6.74)	2.82(1.10,7.22)	2.78(1.05,7.33)	2.40(0.82,7.02)
*P-*value		**0.037**	**0.031**	**0.039**	**0.11**

B1: α-HBDH < 175.90 (n = 106).

B2: α-HBDH≥175.90 (n = 107).

Model 1: adjusted for age and gender.

Model 2: Model 1 further adjusted for BMI, smoker, alcohol, SBP, history of hypertension, dyslipidemia, diabetes and coronary heart disease, hematoma volume, and location.

Model 3: Model 2 further adjusted for ALT, AST, CK, CK-MB, MYO, cTnI, and WBC.

## Discussion

The adverse consequences of cardiac enzymes, particularly α-HBDH in acute stroke, are widely debated. α-HBDH represents LDH1 and LDH2, which are localized in the brain and reticuloendothelial system (16). However, cardiac LDH isoenzymes (LDH1 and LDH2) account for approximately 90% of the total LDH in the human myocardium. α-HBDH is as same as CK-MB, AST, LDH, and creatine kinase in cardiomyocytes. These enzymes contribute to the formation of the myocardial enzyme spectrum, and their levels reflect hemolysis and heart injury (17). In this study, we noticed that high α-HBDH at admission is associated with outcomes in patients with ICH after 1-year follow-up. This relation remained after adjusting for important potential confounders in a multivariable manner.

In our research, we used the median of α-HBDH 175.90 U/L as a cutoff value to classify the groups. The rationality is as follows: First, our study aimed to evaluate the effect of α-HBDH on ICH outcome, so we intended to divide the population into two groups, patients with a relatively low level of the α-HBDH and relatively high level of α-HBDH, and we tried to use the median of α-HBDH. After the statistical analysis, we found that the result had a positive significance. Second, the reference interval of α-HBDH in our laboratory is 72–182 U/L and the median of α-HBDH 175.90 U/L is close to the upper limit, so this declared that the patients of the B2 group had a high or upper limit level of α-HBDH. It was a reasonable design.

There are relatively few published studies about α-HBDH and ICH outcomes in the literature. In comparative clinical trials, α-HBDH appears to be the best marker enzyme for estimating infarct size and assessing the efficacy of reperfusion (18). Lee (13) confirmed the association of α-HBDH with atherothrombotic events after infrainguinal angioplasty with revascularization. This study reported a significant association between high-level α-HBDH and poor outcomes in patients with ICH (*P* < 0.001). At 3 months follow-up, the level α-HBDH in the B2 group was still higher than in the B1 group. We speculated that the elevated levels of α-HBDH in ICH patients with a poor prognosis are caused by systemic inflammatory responses following ICH, which may contribute to myocyte injury, cell death, and brain-heart axis irregularities (19, 20). Myocardial damage can occur after a stroke but the mechanism is still unclear. Chen et al. (21) outlined the brain-heart interaction mechanism, the hypothalamic-pituitary-adrenal (HPA) axis, catecholamine surge, sympathetic and parasympathetic regulation, and the disruption of the blood-brain barrier following stroke. In animal models and humans, research showed that immune responses and inflammatory processes are associated with the pathological cascade that leads to cardiac muscle impairment and heart diseases (22, 23). Proinflammatory cytokines can lead to left ventricle (LV) dysfunction, LV dilation, cardiomyocyte hypertrophy, and death of cardiac myocytes (24). Atherosclerosis, re-infection, heart failure, and hypertrophic cardiomyopathy are attributed to greater ROS levels (25–27). As a result, injury and oxidative stress may play an important role in the brain-heart interaction following ICH. Further research into the protective role after a stroke is required.

Cardiometabolic disturbance has been associated with early mortality and a poor prognosis in patients with stroke (28, 29). A study identified several cardiac complications after 2 days of intracerebral hemorrhage and 3 months of ischemic stroke (6, 30). Another study of more than 1,200 patients found a significant association between troponin positivity and cardio-embolic events, particularly large vessel occlusion (31). After adjusting some confounding factors in our study, we found a correlation between α-HBDH levels, mortality, and recurrent cerebro-cardiovascular events in Models 1 and 2. In Model 3, we concluded an independent association between the α-HBDH and poor functional outcomes even after adjusting for age, sex, smoking, BMI, alcohol, baseline hematoma site and volume, and other cardiac enzymes. Despite our differences with other studies, we justify our findings by citing a small patient population, the follow-up time and the low severity of intracerebral hemorrhage in our patients.

Although the relation between LDH isoenzymes and stroke complications has yet to be discovered, several plausible reasons have been suggested. LDH is a valuable inflammatory stress prognostic marker (32, 33). Vascular injury, atherosclerosis, and atheroma volatility are linked to inflammation, leading to pathological death and a poor prognosis (34–36). In addition, LDH can be found in different organs. Its serum level rises in the presence of many diseases (37–39). Thus, lactate dehydrogenase, a substitute for the extent of the injury to different organs, could serve as a valuable indicator of stroke-related adverse events. Further research on the mechanisms is required.

It should be pointed out that this is a single-center study. The enrolled patients only represented the northern area around Beijing. Table 1 showed that the median NIHSS score for both groups was 7, which meant the whole cohort had a moderate stroke. On one hand, this may be one of the reasons for the low mortality and HE occurrence in the cohort. On the other hand, the patients may have a relatively long survival time. Thus, we need to find early potential biomarkers to predict the outcomes to elevate the quality of life at an early stage.

It is crucial to consider the study's relative strengths and weaknesses. The study's samples were representative of North China's adult population, and the study employed a retrospective design. The α-HBDH was an essential predictor of ICH outcomes. This finding suggests that α-HBDH is the same as LDH for predicting poor outcomes.

Our research has certain limitations: (1) The study was a single-center study. (2) We only looked at the cardiac serum index. We did not check the electrocardiogram or the cardiac doppler ultrasound for actual cardiac changes. (3) Large cohort studies will be required to validate the predictive value of α-HBDH in ICH. (4) The role and underlying mechanisms of α-HBDH in predicting adverse outcomes in ischemic stroke and other cardiovascular diseases need to be further investigated in more clinical trials.

Of note, this study documented that high α-HBDH levels at admission are correlated with poor outcomes among patients with ICH. Patients of ICH with a high level of α-HBDH in their blood may be at greater risk for complications.

## Conclusion

Our findings show that patients with ICH have a poor prognosis when their serum α-HBDH levels are elevated. It is possible to use α-HBDH as an early indicator to predict the outcomes of patients with ICH.

## Data availability statement

The raw data supporting the conclusions of this article will be made available by the authors, without undue reservation.

## Ethics statement

The studies involving human participants were reviewed and approved by the Institutional Review Board (IRB) of Beijing Tiantan Hospital, Capital Medical University. The patients/participants provided their written informed consent to participate in this study.

## Author contributions

All authors listed have made a substantial, direct, and intellectual contribution to the work and approved it for publication.

## Funding

This study was supported by the Beijing Hospital Authority Clinical Medicine Development of Special Funding (Grant No. ZYLX202108) and the Research incubation fund of Capital Medical University (No. PYZ2017067).

## Conflict of interest

The authors declare that the research was conducted in the absence of any commercial or financial relationships that could be construed as a potential conflict of interest.

## Publisher's note

All claims expressed in this article are solely those of the authors and do not necessarily represent those of their affiliated organizations, or those of the publisher, the editors and the reviewers. Any product that may be evaluated in this article, or claim that may be made by its manufacturer, is not guaranteed or endorsed by the publisher.
